# Role of *Geitlerinema* sp. DE2011 and *Scenedesmus* sp. DE2009 as Bioindicators and Immobilizers of Chromium in a Contaminated Natural Environment

**DOI:** 10.1155/2015/519769

**Published:** 2015-06-17

**Authors:** Laia Millach, Antoni Solé, Isabel Esteve

**Affiliations:** Departament de Genètica i Microbiologia, Facultat de Biociències, Universitat Autònoma de Barcelona, Bellaterra, Cerdanyola del Vallès, 08193 Barcelona, Spain

## Abstract

The aim of this work was to study the potential of the two phototrophic microorganisms, both isolated from Ebro Delta microbial mats, to be used as bioindicators and immobilizers of chromium. The results obtained indicated that (i) the Minimum Metal Concentration (MMC) significantly affecting Chlorophyll *a* intensity in *Geitlerinema* sp. DE2011 and *Scenedesmus* sp. DE2009 was 0.25 *µ*M and 0.75 *µ*M, respectively, these values being lower than those established by current legislation, and (ii) *Scenedesmus* sp. DE2009 was able to immobilize chromium externally in extracellular polymeric substances (EPS) and intracellularly in polyphosphate (PP) inclusions. Additionally, this microorganism maintained high viability, including at 500 *µ*M. Based on these results, we postulate that *Geitlerinema* sp. DE2011 and *Scenedesmus* sp. DE2009 are good chromium-indicators of cytotoxicity and, further, that *Scenedesmus* sp. DE2009 plays an important role in immobilizing this metal in a contaminated natural environment.

## 1. Introduction

Metal contamination is a serious environmental problem that affects life forms and changes the natural microbiota of aquatic ecosystems. Currently, metals are released from natural and anthropogenic sources (e.g., industry, transport, fossil fuel combustion, the mining industry, and agriculture) into natural aquatic environments [1]. These metals are accumulated in waters, sediments, and biota, generating resistance in microorganisms that leads to environmental and public health problems. To study and predict the effects and removal of heavy metals on different ecosystems, nematode [2], plants [3], and algae [4], among others, have been used. Cyanobacteria and algae are particularly very abundant in aquatic ecosystems, playing an important role in primary production in rivers and their deltas, where metals very often accumulate.

The Ebro River is 928 km long, flows from the north of the Iberian Peninsula to the Mediterranean Sea, and drains an area of 85,000 km^2^ approximately. The Ebro Delta, located at the outfall of the Ebro River, is the second most important wetland in Spain after Guadalquivir River marshes and the second one of the Mediterranean area after the Camargue (France). The Ebro Delta is also considered the third largest delta in the Mediterranean with a 320 km^2^ triangular surface and it is located at the northeastern coastline of the Iberian Peninsula (0°35′E–0°56′E; 40°33′N–40°47′N) [5]. In 1983, some of the most outstanding natural areas of the delta were included in the Ebro Delta Natural Park (*Parc Natural del Delta de l'Ebre*) because of its ornithological importance, as well as for other geological, biological, economic, and cultural aspects [6].

Microbial mats, developed in water-sediment interfaces, are formed by multilayered benthic microbial communities that are distributed along vertical microgradients of different physical-chemical parameters. These ecosystems are widely distributed around the world in different extreme environments, such as lakes [7], marine waters [8], and cold waters [9], among others. Ebro Delta microbial mats are formed by different microorganisms; principally cyanobacteria and microalgae are the most abundant prokaryotic bacteria located mainly in the upper layers of microbial mats [10]. These microbial mats receive waters and contaminants, including heavy metals dragged by the River Ebro into its estuary (delta). For this reason, in the last few years, our work group has isolated various microorganisms of this ecosystem and has developed several methods, in particular Confocal Laser Scanning Microscopy (CLSM), to determine their capacity to tolerate or resist metals, as well as evaluate the effect of these* in vivo* at both cell and population levels. These methods used for the* in vivo* study of phototrophic microorganisms have led to obtaining quantitative results more quickly. This is mainly due to the minimal necessary manipulation of the specimens, and since these emit natural fluorescence, they do not require staining protocols. Furthermore, the majority of works have evaluated the effect of lead and copper toxicity in isolated microorganisms [11] and the capacity of various microorganisms to uptake these metals extra- and/or intracellularly using Scanning Electron Microscopy (SEM) and Transmission Electron Microscopy (TEM), both coupled to an Energy Dispersive X-Ray (SEM-EDX and TEM-EDX) [12].

However, the role that microorganisms play in this same habitat on chromium detoxification is still unknown. Chromium can exist in the environment as Cr(III) or Cr(VI) [13, 14] and particularly in the Cr(VI) form is extremely toxic, mutagenic, and carcinogenic. In the environment, chromium is introduced as the by-product of industries [15] and phosphate fertilizers [16]. In highly contaminated habitats [17, 18], the reduction of Cr(VI) to Cr(III) is an effective method of Cr(VI) detoxification. Nevertheless, the immobilization efficiency of Cr(III) is still unclear and different reports suggest that soluble organo-Cr(III) complexes are present in various chromate-reducing bacterial systems [19, 20]. The permanence of soluble forms of Cr(III) causes a serious problem, since they can be reoxidized to Cr(VI). It is for this reason that there is great interest in studying the immobilization of Cr(III) in pilot-scale experiments [21].

Nowadays, there is little information on this process in the natural environment, where the levels of contamination by chromium are very low, as in the River Ebro (<2 *μ*g L^−1^ Cr, according to data from the Ebro Hydrographic Association, in the last 10 years). In these cases, although the same probably occurs, the Cr(VI) is biotransformed to Cr(III), and this can remain in ecosystems, immobilized or not, and could have a toxic effect on life forms. Likewise, very little is known about the role of indigenous microorganisms in these natural environments with low levels of chromium and also with a prolonged permanence of the metal in the ecosystem.

The aim of this work is to determine the role of* Geitlerinema* sp. DE2011 and* Scenedesmus* sp. DE2009, both isolated from Ebro Delta microbial mats, as bioindicators and immobilizers of chromium and, additionally, to analyse the effect of this metal on their biomass and cellular viability.

## 2. Material and Methods

### 2.1. Microorganisms and Culture Conditions

*Geitlerinema* sp. DE2011 (cyanobacterium) and* Scenedesmus* sp. DE2009 (microalga) were isolated from Ebro Delta microbial mats (Tarragona), Spain. Isolation and purification of the isolates were performed by dilution and plating of microbial mats samples. Isolated microorganisms were grown in liquid mineral Pfennig medium [22] in 100 mL flasks. Cultures were exposed and maintained at 27°C in a growth chamber (Climas Grow 180, ClimasLab, Barcelona) under continuous illumination with a light intensity of 3.5 *μ*E m^−2^ s^−1^ for the cyanobacterium and 10 *μ*E m^−2^ s^−1^ for the microalga, provided by cold white fluorescence lights. These cultures were used as control in all the experiments performed.

### 2.2. Preparation of Chromium Stock Solution

The stock solution of chromium was prepared by dissolving Cr(NO_3_)_3_ (Sigma-Aldrich, Bellefonte, PA, US) in deionized Milli-Q water at the concentration of 1 mM Cr(III) and sterilized by filtration in Millex-GP 0.2 *μ*m filters (Millipore, USA). Working concentrations of Cr(III) were obtained by serial dilution. This solution was stored in the dark at 4°C.

### 2.3. Pigment Analysis of the Strains Using Confocal Laser Scanning Microscopy

The tolerance and the* in vivo* effect of chromium on cultures of* Geitlerinema* sp. DE2011 and* Scenedesmus* sp. DE2009 were determined by  *λscan* function of CLSM (CLSM Leica TCS SP5; Leica Heidelberg, Germany). Moreover, in order to evaluate the effect of chromium on the biomass and viability of* Scenedesmus* sp. DE2009, a modification of the FLU-CLSM-IA (Fluorochrome-CLSM-Image Analysis) method described by Puyen et al. [23] was used.

#### 2.3.1. *λscan* Function

Cultures of* Geitlerinema* sp. DE2011 and* Scenedesmus* sp. DE2009 were contaminated at different Cr(NO_3_)_3_ concentrations: 0.025, 0.050, 0.1, 0.25, 0.50, 0.75, 1, and 5 *μ*M for the cyanobacterium DE2011 and 0.25, 0.50, 0.75, 1, 5, 10, 15, and 25 *μ*M for the microalga DE2009. All experiments were performed for 9 days under the same conditions mentioned in Section 2.1.

Pigment analysis was realized by the  *λscan* function of CLSM. This technique provides information on the state of the photosynthetic pigments of phototrophic microorganisms on the basis of the emission wavelength region and the fluorescence intensity emitted (autofluorescence). Each image sequence was obtained by scanning the same *xy* optical section throughout the visible spectrum. Images were acquired at the *z* position at which the fluorescence was maximal, and acquisition settings were constant throughout each experiment. The sample excitation was carried out with an Argon Laser at 488 nm (*λ*exe 488) with a *λ* step size of 3 nm for an emission wavelength between 550 and 748 nm.

In order to measure the mean fluorescence intensity (MFI) of the *xyλ* data sets, the Leica Confocal Software (Leica Microsystems CMS GmbH) was used. In these confocal images the pseudocolour palette 4 was selected, where warm colours represented the maximum intensities and cold colours represented the low intensities of fluorescence. The regions-of-interest (ROIs) function of the software was used to measure the spectral signature. For each sample, 70 ROIs of 1 *μ*m^2^ taken from cells were analysed.

This method allowed us to evaluate the physiological state of the phototrophic microorganisms at single-cell level, considering changes in the spectrum of Chlorophyll* a* (Chl* a*) used as a marker. For this purpose, the state of pigments was considered by means of the Maximum Intensity Fluorescence (MIF) signal detected at 661 nm (Chl* a*) for* Geitlerinema* sp. DE2011 and 685 nm for* Scenedesmus* sp. DE2009 (Chl* a*).

#### 2.3.2. FLU-CLSM-IA Modified Method

To determine the effect of chromium on biomass and cellular viability of* Scenedesmus* sp. DE2009 cultures, experiments at different Cr(NO_3_)_3_ concentrations, 0.75, 25, 100, 200, and 500 *μ*M, were performed for a period of 9 days under the same conditions mentioned in Section 2.1 following a modification of the FLU-CLSM-IA method [23]. This method combines the use of specific fluorochrome, the CLSM microscope, and the* ImageJ v1.48s* software.

**Figure 1:**
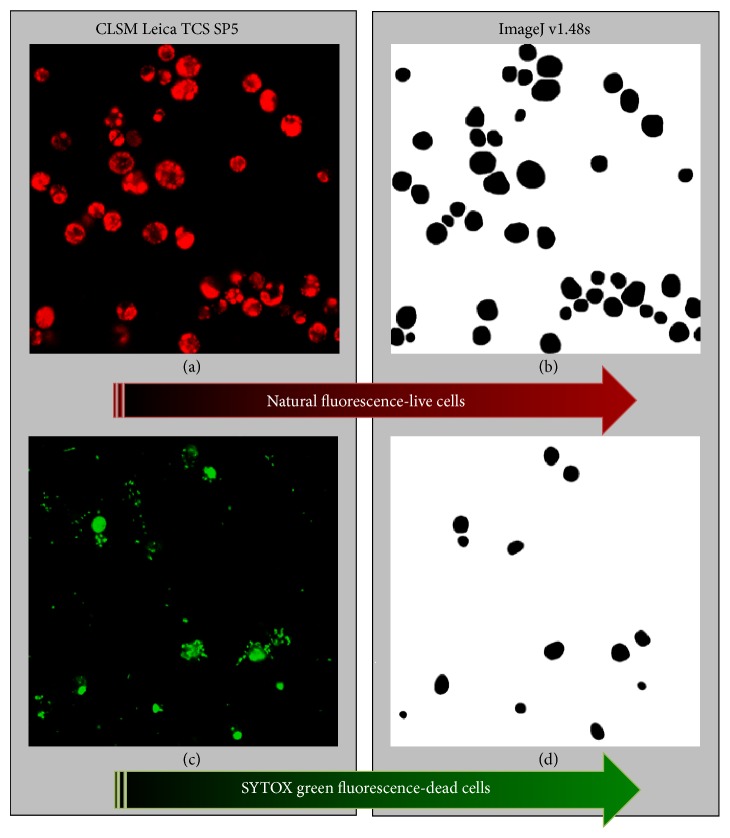
*xyz* CLSM optical sections (a) and (b) and their corresponding binary images of live (b) and dead (d)* Scenedesmus* sp. DE2009 cells analysed using the modified FLU-CLSM-IA method.

In this study,* Scenedesmus* sp. DE2009 autofluorescence (emission at 616–695 nm) and SYTOX Green Nucleic Acid Stain fluorescence (emission at 520–580 nm; Invitrogen, Life Technologies) were used simultaneously as markers for live and dead cells, respectively, in a simple dual-fluorescence viability assay [24]. Both the red and green fluorescence signals were captured separately in a* sequential scan* process in two channels from each same *xyz* optical section (Figures 1(a) and 1(c)).

In order to differentiate between living and dead cells, red (live cells) and green (dead cells) pseudocolors were used and 20 red and green confocal images were acquired from every culture of* Scenedesmus* sp. DE2009 to determine the biomass and cellular viability at each Cr-concentration.

The CLSM images were transformed to binary images (black/white) applying fluorescence threshold values of 30 (red pixels) and 35 (green pixels) by means of the* ImageJ v1.48s* software (Figures 1(b) and 1(d)). To minimize the background detected in every pair of images a smoothing filter was used.

To obtain biovolume values, the Voxel Counter plug-in was applied to these filtered images [25]. This specific application calculates the ratio between the thresholded voxels (red and green fluorescent voxel counts) to all voxels from every binary image analysed. The biovolume value (volume fraction) was finally multiplied by a conversion factor of 310 fgC *μ*m^3^ to convert it to biomass [26].

### 2.4. Ascertaining Chromium Immobilization through Electronic Microscopy Techniques

With the aim of determining whether* Geitlerinema* sp. DE2011 and* Scenedesmus* sp. DE2009 could immobilize metals extra- and intracellularly, cells from cultures growing with and without chromium were analysed by EDX coupled to SEM and TEM.

#### 2.4.1. Scanning Electron Microscopy and Energy Dispersive X-Ray Analysis

Phototrophic microorganisms cultures were contaminated at different Cr(NO_3_)_3_ concentrations, 1, 5, 10, 25, 50, 100, and 200 *μ*M Cr(III), and incubated under the same conditions as mentioned above for a period of 9 days.

For SEM analysis, cultures were filtrated in Nuclepore polycarbonate membranes (Whatman, Ltd.) and then were fixed in 2.5% glutaraldehyde diluted in Millonig phosphate buffer (0.1 M pH 4) at 4°C for 2 hours and washed four times in the same buffer, dehydrated in increasing concentrations of ethanol (30%, 50%, 70%, 90%, and 100%), and dried by critical-point (CPD 030 Critical Point Drier, BAL-TEC GmbH, 58579 Schalksmühle). Finally, samples were mounted on aluminium metal stubs and coated with a 5 *μ*m gold layer (K550 Sputter Coater, Emitech, Ashford, UK) for better image contrast. A Zeiss EVOMA 10 scanning electron microscope (Carl Zeiss NTS GmbH, Oberkochen, Germany) was used to view the images.

For EDX microanalysis, cells were homogenously distributed and filtered on polycarbonate membrane filters. These filters were fixed, dehydrated, and dried by critical-point drying and then coated with gold. An EDX spectrophotometer Link Isis-200 (Oxford Instruments, Bucks, England) coupled to the microscope operating at 20 kV was used. Finally, EDX-SEM spectra from individual cells were obtained.

#### 2.4.2. Transmission Electron Microscopy and Energy Dispersive X-Ray Analysis

TEM was used in order to observe the ultrastructure of the phototrophic microorganisms and TEM-EDX to assess whether* Geitlerinema* sp. DE2011 and* Scenedesmus* sp. DE2009 were able to bioaccumulate chromium intracellularly. So, cyanobacterium DE2011 and the microalga DE2009 were contaminated with 200 *μ*M Cr(NO_3_)_3_ for a period of 9 days. Culture conditions were the same as described for SEM.

For TEM analysis, samples were fixed in 2.5% glutaraldehyde diluted in Millonig phosphate buffer (0.1 M pH 4) at 4°C for 2 hours and washed four times (15 min) in the same buffer at 4°C. Then samples were postfixed in 1% OsO_4_ at 4°C for 2 hours, washed in the same buffer, and centrifuged in order to obtain a pellet. They were then dehydrated in a graded series of acetone (50%, 70%, 90%, 95%, and 100%) and embedded in Spurr's resin. Once the samples were included in the resin, ultrathin sections (70 nm), obtained with a Leica EM UC6 Ultramicrotome (Leica Microsystems, GmbH, Heidelberg, Germany), were mounted on carbon-coated titanium grids and stained with uranyl acetate and lead citrate. Samples were viewed in a Hitachi H-7000 transmission electron microscope (Hitachi Ltd., Tokyo, Japan).

For EDX microanalysis, sections 200 nm thick were also stained with uranyl acetate and mounted on carbon-coated titanium grids. Samples were analysed with an EDX spectrophotometer Link Isis-200 (Oxford Instruments, Bucks, England) coupled to a Jeol Jem-2011 (Jeol Ltd., Tokyo, Japan) operating at 20 kV. Finally, EDX-TEM spectra from individual cells were obtained.

### 2.5. Statistical Analysis

Statistical analyses were carried out by one-way analysis of variance (ANOVA) and Tukey and Bonferroni's comparison* post hoc* tests. Significant differences were accepted at *P* < 0.05. The analyses were performed using IBM SPSS Statistics software (version 20.0 for Windows 7).

## 3. Results and Discussion

### 3.1. Morphological Characteristics of* Geitlerinema* sp. DE2011 and* Scenedesmus* sp. DE2009

The phototrophic microorganisms, isolated from Ebro Delta microbial mats, were identified as* Geitlerinema* sp. DE2011 [27] and* Scenedesmus* sp. DE2009 [12] by molecular biology methods. Both microorganisms are very abundant in Ebro Delta microbial mats and play an important role in the stabilization of deltaic sediments.

*Geitlerinema* sp. DE2011 is a cyanobacterium, which forms individual filaments, sometimes densely packed and surrounded by a sheath. Cells from filaments vary in size from 3.13 to 3.75 *μ*m. On the other hand,* Scenedesmus* sp. DE2009 is a microalga, which like* Geitlerinema* sp. DE2011 forms a consortium with different heterotrophic bacteria. The microalga cells are spherical, with a diameter of 7–9 *μ*m and their chloroplasts are distributed laterally in the cell.

### 3.2. Chromium Tolerance in Phototrophic Microorganisms

**Figure 2:**
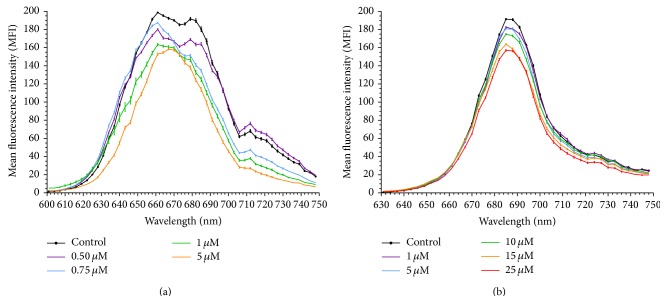
*λscan* plots of* Geitlerinema* sp. DE2011 (a) and* Scenedesmus* sp. DE2009 (b) contaminated with a wide range of chromium concentrations.

In order to calculate the Minimum Metal Concentration (MMC) that significantly affects pigment intensity in* Geitlerinema* sp. DE2011 and* Scenedesmus* sp. DE2009, two experiments were performed. In the preliminary experiment, a wide range of chromium concentrations was assayed. Displacement of the fluorescence peak was observed only in* Geitlerinema* sp. DE2015 from 661 nm (MIF) towards to 670 nm, at maximum Cr-concentration assayed (5 *μ*M fluorescence spectrum). In both cases, highly statistically significant differences (*P* < 0.05) were found between the control and all the concentrations tested (Figures 2(a) and 2(b)).

**Figure 3:**
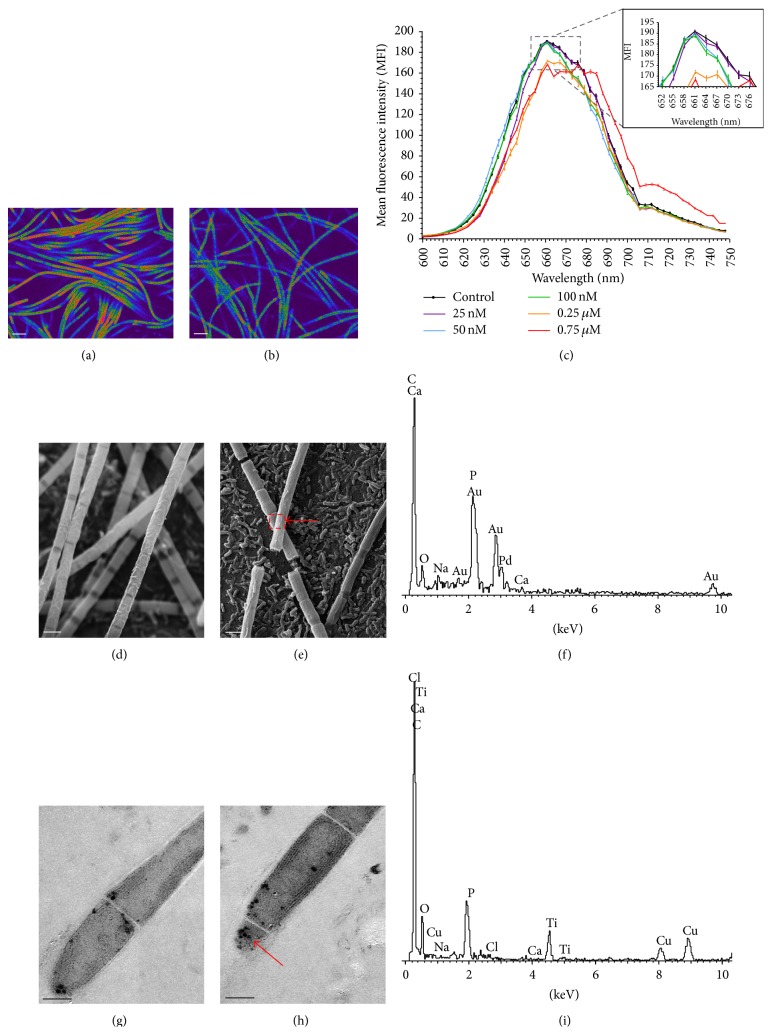
CLSM images of control (a) and chromium contaminated (b) cultures of* Geitlerinema* sp. DE2011 (scale bars represent 10 *µ*m) and *λscan* plot (c). SEM images of control (d) and 200 *µ*M chromium contaminated (e) cultures. Scale bars represent 2 *µ*m. Contaminated EDX spectrum (f). TEM images of control (g) and 200 *µ*M chromium contaminated (h) cultures. Scale bars represent 1 *µ*m. Contaminated EDX spectrum (i).

**Figure 4:**
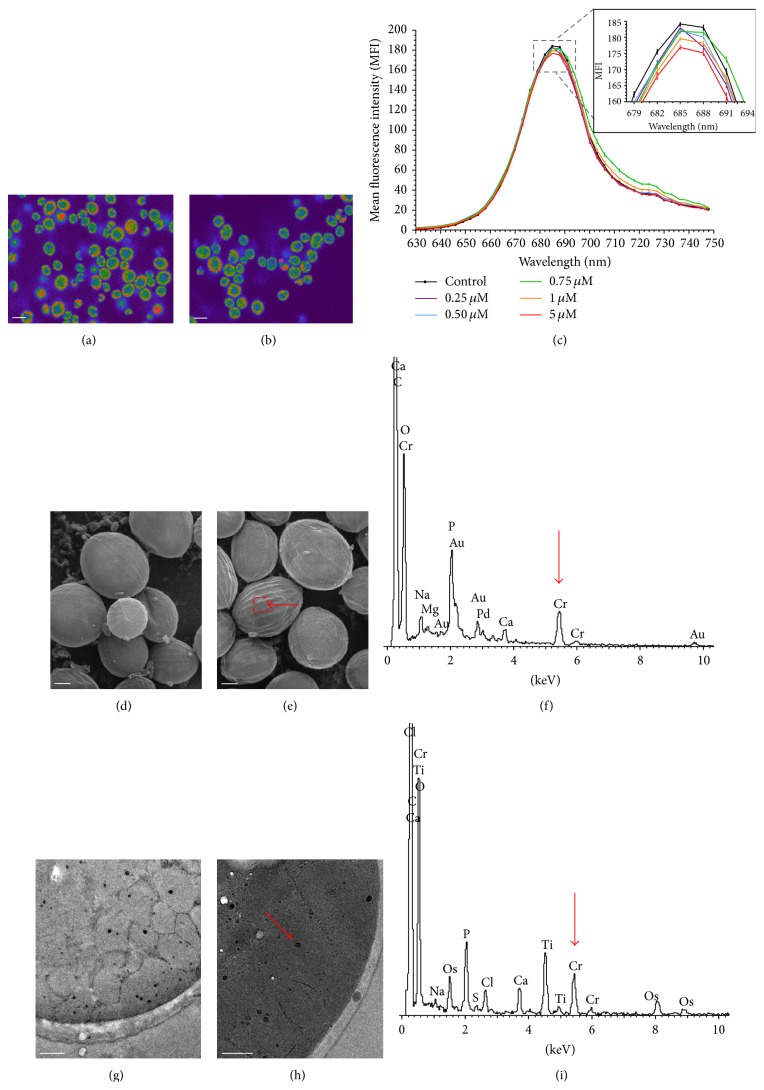
CLSM images of control (a) and chromium contaminated (b) cultures of* Scenedesmus* sp. DE2009 (scale bars represent 10 *µ*m) and *λscan* plot (c). SEM images of control (d) and 200 *µ*M chromium contaminated (e) cultures. Scale bars represent 2 *µ*m. Contaminated EDX spectrum (f). Arrow indicates the main Cr peak at 5.4 keV. TEM images of control (g) and 200 *µ*M chromium contaminated (h) cultures. Scale bars represent 1 *µ*m. Contaminated EDX spectrum (i). Cr peaks are indicated by arrows.

For this reason, a second experiment was carried out on* Geitlerinema* sp. DE2011 with lower doses from 25 nM to 0.75 *μ*M Cr(III). The *xyz* optical sections of this microorganism, corresponding to the autofluorescence detected in control and contaminated cultures, were shown in Figures 3(a) and 3(b). The results indicated that the MMC of chromium (when compared with the control) that significantly (*P* < 0.05) affected the intensity of the pigment (Chl* a*) of* Geitlerinema* sp. DE2011 was 0.25 *μ*M Cr. An analogous experiment to that mentioned above was performed with lower doses from 0.25 *μ*M to 5 *μ*M Cr(III) on cultures of* Scenedesmus* sp. DE2009. The autofluorescence detected in control and contaminated cultures was shown in Figures 4(a) and 4(b). In this case, the MMC that significantly (*P* < 0.05) affected the intensity of the pigment in* Scenedesmus* sp. DE2009 was 0.75 *μ*M Cr, and therefore this microorganism was more tolerant to chromium than* Geitlerinema* sp. DE2011 (0.25 *μ*M Cr).

On the other hand, the *λscan* plots graphs of both microorganisms indicated how the MIF peak (Chl* a*) decreased while the Cr-concentration increased following mainly the same pattern as the control culture (Figures 3(c) and 4(c)). These results are in agreement with those obtained by different authors, which demonstrated, in* Scenedesmus obliquus* and* Nostoc muscorum*, respectively, that metal stress results in direct inactivation of the photosystem II (PS II) reaction center and consequently a decrease of Chlorophyll* a* fluorescence intensity (F_685_) [28, 29]. Furthermore, other authors have demonstrated that in response to varying physical-chemical parameters photosynthetic microorganisms undergo changes in their physiological characteristics, mainly changing the quality and concentration of their light-harvesting pigments [30].

It is worth highlighting that the MMC values obtained were below the level permitted in continental surface waters (50 *μ*g L^−1^ Cr) (in accordance with the Directive 2008/105/CE of the European Parliament and the Council on Environmental Quality Standards in the field of Water Policy, transposed into Spanish law “Real Decreto 60/2011, Anexo II”), which demonstrated that both microorganisms should be considered as good indicators of cytotoxicity.

### 3.3. Metal Immobilization in Phototrophic Microorganisms

Cr-contaminated cultures of* Geitlerinema* sp. DE2011 were analysed by SEM-EDX and chromium was not detected in the extracellular polymeric substances (EPS) (Figures 3(d), 3(e), and 3(f)). Nevertheless, in the contaminated samples of* Scenedesmus* sp. DE2009, the results confirmed that the microalga had the ability to sequester chromium in the EPS (Figures 4(d), 4(e), and 4(f)). Different parts of the filter were also tested as a control in all samples, to be sure that chromium was retained only in cells.

Both microorganisms have dense EPS envelopes, which explain the external uptake of heavy metals. Various authors have suggested that the overall negative charge of EPS may be essential for sequestering metal cations that are necessary for cell growth but present at low concentrations in their surroundings and/or preventing the direct contact between the cells and toxic heavy metals dispersed in the environment [31]. The functions of EPS in metal uptake are known, but other roles have been proposed for these polymers, such as protection against dehydration or UV radiation, biomineralization, phagocytosis, and adhesion capacity to the surrounding substrate [32].

Although* Geitlerinema* sp. DE2011 gave a negative result for chromium uptake, in previous studies it has been shown that this cyanobacterium was able to capture lead and copper extracellularly [27]. These differences in metal immobilization were probably due to the fact that the same microorganism can capture distinct metals using different functional groups in the EPS. In accordance with studies carried out by Ozturk et al. [33] an increase in uronic acid, glucuronic acid, and galacturonic acid content was shown in the EPS of* Synechocystis* sp. BASO671 cultures contaminated by chromium. In addition, Çelekli et al. [34] also confirmed that specific anionic groups played a significant role in the biosorption of Cd^2+^ by* Scenedesmus quadricauda* var.* longispina*.

On the other hand, TEM micrographs of the ultrathin sections of* Geitlerinema* sp. DE2011 and* Scenedesmus* sp. DE2009 growing at 200 *μ*M Cr showed abundant high electron dense intracytoplasmic inclusions of different sizes in their cytoplasm identified as polyphosphate inclusions (PP) (Figures 3(h) and 4(h)). In many cases, similar inclusions have been found when cells are grown in adverse culture conditions [35]. Chromium was not detected internally in* Geitlerinema* sp. DE2011 or* Scenedesmus* sp. DE2009 in control cultures (Figures 3(g) and 4(g)).

The results obtained through EDX analysis of the inclusions demonstrated that* Geitlerinema* sp. DE2011 did not have the capacity to accumulate chromium as no Cr peak was detected (Figure 3(i)). In contrast to this, a significant Cr peak was detected in* Scenedesmus* sp. DE2009, demonstrating that this microorganism was able to immobilize this metal internally in PP inclusions (Figure 4(i)). These results agree with studies of Goldberg et al. [36], which suggested that this kind of inclusions has a detoxifying effect and a large affinity by sequestering heavy metals. In general, algae seem to be more effective than cyanobacteria in capturing heavy metals [37, 38] and, as has been shown in this work,* Scenedesmus* sp. DE2009, due to its ability to capture chromium both extra- and intracellularly, probably plays an important role in chromium detoxification in Ebro Delta microbial mats.

### 3.4. Effect of Chromium on Biomass and Cellular Viability of* Scenedesmus* sp. DE2009

For this objective, previously, the red and green fluorescent voxels counts were measured as mentioned in Section 2.3.2. The red voxels (live cells) ranged from 162097 ± 9220 (control experiment) to 143390 ± 6638 (at 500 *μ*M) and the green voxels (dead cells) varied from 23450 ± 1822 (control experiment) to 32113 ± 2277 (at 500 *μ*M). The conversion of this data into biomass values made it possible to observe that the live biomass slightly decreased from 47.92 ± 2.73 mgC cm^−3^ in the control culture to 42.39 ± 1.96 mgC cm^−3^ at 500 *μ*M Cr.

**Figure 5:**
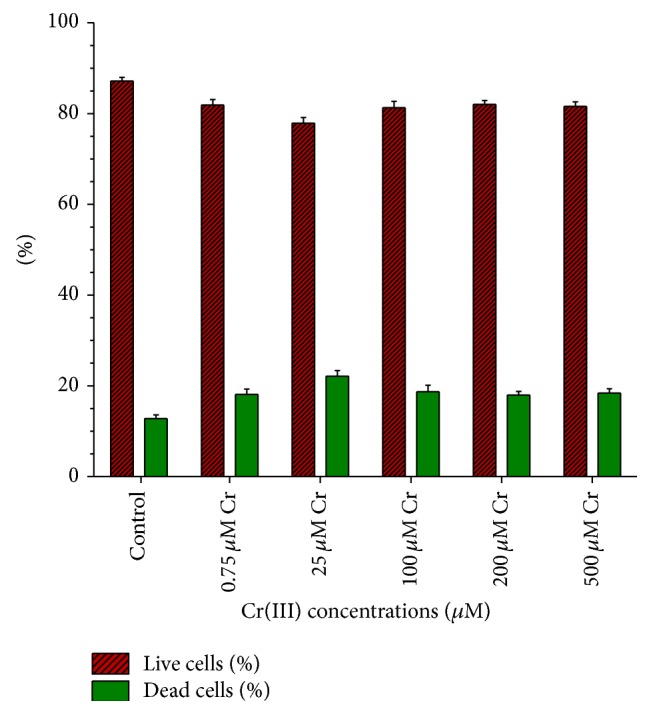
Percentages of live and dead* Scenedesmus* sp. DE2009 cells at different Cr(III) concentrations. The bars indicate the Standard Error of the Means (S.E.M.).

The changes in viability were shown in Figure 5. These results were expressed as the percentages (%) of live cells and dead cells for each contaminated culture. These values showed low significant differences (*P* < 0.05) for all of them compared to the control culture, which indicated a slight effect of the metal in the viability of* Scenedesmus* sp. DE2009. However, there were no significant differences (*P* < 0.05) between the various concentrations tested, with the percentage of viable cells in all the Cr-concentrations tested remaining stable.

Thus, on comparing the growth of* Scenedesmus* sp. DE2009 in control culture and the maximum tested concentration (500 *μ*M), it was observed that in the control experiment live cells represented 87.19% and dead cells 12.81%, and in the contaminated culture live cells represented 81.61% and dead cells 18.39% (Figure 5). These results confirmed that a high level of viability of the microalga is maintained, even at the highest concentration of chromium tested.

## 4. Conclusions

The results obtained in this paper lead to the conclusion that* Scenedesmus* sp. DE2009 is more tolerant to chromium than* Geitlerinema* sp. DE2011 and that both microorganisms could be considered as good indicators of chromium toxicity in low contaminated natural ecosystems.

On the other hand,* Scenedesmus* sp. DE2009 maintains an elevated biomass and viability at high Cr-concentrations and also has the ability to capture chromium extracellularly in EPS and intracellularly in PP inclusions, which demonstrates its capacity to immobilize this metal.
